# Comparison of Program-centric vs Student-centric National Resident Matching Algorithms

**DOI:** 10.1001/jamanetworkopen.2021.13769

**Published:** 2021-06-16

**Authors:** Briance Mascarenhas, Kartikeya S. Puranam, Michael N. Katehakis

**Affiliations:** 1 School of Business–Camden, Rutgers University, Camden, New Jersey; 2 Rutgers Business School, Rutgers University, Newark, New Jersey

## Abstract

**Importance:**

The current program-centric algorithm for the National Resident Matching Program (NRMP) primarily uses the program’s ranking of students to determine a match. Concerns that the existing algorithm favors programs over students, recent findings that the program’s ranking of applicants is not associated with resident performance, and disruptions of existing screening methods and metrics have prompted reevaluation of the current algorithm relative to a student-centric algorithm, in which student ranking of programs is primary and program ranking of students is secondary.

**Objective:**

To compare program-centric and student-centric algorithms for the NRMP participants.

**Design, Setting, and Participants:**

This cross-sectional study used randomized computer-generated data reflecting the NRMP match for 2018, 2019, and 2020, capturing more than 50 000 students and more than 4000 programs in 23 specialties, to compare the 2 algorithms.

**Exposures:**

The same simulated students, programs, and rankings were exposed to the 2 algorithms, running 2300 simulations in the overall analysis and 1000 simulations in each of 23 specialties.

**Main Outcomes and Measures:**

The percentage of students who did and did not match, the percentage of students who matched to their top-ranked and top-5–ranked programs, and the program’s rank of the last student matched per position were examined.

**Results:**

The 2 algorithms were not different in percentage of students matched overall (eg, for 2020, program-centric: 59% [95% CI, 57%-61%]; student-centric: 58% [95% CI, 56%-60%]; *P* = .73). The student-centric algorithm, relative to the program-centric algorithm, matched a significantly higher percentage of students to their first-ranked program (eg, for 2020, 50% [95% CI, 48%-52%] vs 14% [95% CI, 13%-15%]; *P* < .001) and to their top-5–ranked programs (eg, for 2020, 60% [95% CI, 58%-62%] vs 46% [95% CI, 44%-48%]; *P* < .001). However, the last position was filled with students who had lower program rankings in the student-centric algorithm vs the program-centric algorithm (2 [95% CI, 1-2] vs 8 [95% CI, 6-10]; *P* < .001).

**Conclusions and Relevance:**

In this study, the 2 algorithms were not different in the percentage of students matched overall. However, the student-centric algorithm matched a significantly higher percentage of students to their preferred programs. The program-centric algorithm was associated with a lower program’s last matched student rank. Further research is needed on the algorithms’ associations with cost and time demands in the match, postmatch resident and program performance, and fit with a changing environment.

## Key Points

**Question:**

How does the current program-centric algorithm for the National Resident Matching Program (NRMP) compare with a student-centric algorithm?

**Findings:**

In this cross-sectional study of randomized computer-generated data corresponding to the NRMP match for 2018, 2019, and 2020 among more than 50 000 student applicants and 4000 programs in 23 specialties, the 2 algorithms did not differ in percentage of students matched. The student-centric algorithm, relative to the program-centric algorithm, matched a significantly higher percentage of students to their first-ranked program and to their top-5–ranked programs; however, the last position was filled with students who had lower program rankings in the student-centric algorithm vs the program-centric algorithm.

**Meaning:**

These findings suggest that research is needed on these 2 algorithms’ resource demands as well as ensuing resident and program performance.

## Teaser

This cross-sectional study of randomized computer-generated data compares program-centric and student-centric algorithms for the National Resident Matching Program.

## Introduction

The National Resident Matching Program (NRMP) has grown to serve more than 50 000 student applicants to more than 30 000 positions in more than 4000 medical residency programs. Programs invite selected student applicants for interviews. Interviewed students then rank programs by preference and vice versa. Programs and students submit their rank-ordered lists to the NRMP for possible matching through its algorithm.

The process is exhausting for students and programs.^1^ Students have to apply, be selected for interviews, schedule interviews, manage and pay for travel, interview, and submit postinterview rankings, in addition to their ongoing academic and clinical work. Similarly, residency program directors and other administrators face a growing number of applicants to be reviewed, invited, interviewed, and rank ordered.^1,2^ Concerns and debates have arisen regarding the matching process.^3^ Surveys of 206 program directors and 314 students participating in the match reveal that many program directors are confused about how the match actually works and feel it is too expensive and time-consuming for the applicants and programs, while most applicants (60%) perceive that the process needs to be changed.^4^ The NRMP favors programs over students, who are not informed of this bias.^5^ A counterargument has been that since students received higher-ranked matches than programs, students are not treated unfairly.^6^ Nonetheless, several groups, including the American Medical Student Association, the Public Citizen’s Health Research Group, and the Medical Student Section of the American Medical Association, argue that the matching algorithm is biased toward residency programs at the expense of students and seek an algorithm that is more favorable to applicants.^7^ The match process has been legally challenged as uncompetitive, but Congress deemed that the process is ”highly efficient, pro-competitive, and longstanding.”^8^

Several attempts have tried to improve the residency match process for applicants and residency programs.^9^ Some proposals have prompted radical change to residency matching. There has been a call for a modern, free-market approach to residency matching in which hospitals and students negotiate directly year-round.^10^ Such a proposal would allow competent students to apply and fill positions at any point during the year, which could better suit applicants, programs, and the public. Such a proposal involves substantial changes among many stakeholders, but what are needed are “other more immediate practical solutions to the issues with the Match.”^11^

The matching process in the current program-centric algorithm starts by considering students and programs in order of student preference and then matches to the program are decided by the program’s rankings of students. This algorithm was adopted after heated debate and concerns raised regarding how much the former (pre-1998) matching algorithm was biased toward residency programs at the expense of applicants and interest in whether it would be feasible to replace that algorithm with something more favorable to applicants.^7^

However, the revised program-centric algorithm resulted in few match changes—less than approximately 1 in 1000 students or approximately 16 students total—relative to the previous algorithm.^7^ It has been posited that there would be little difference between a program-optimal algorithm and a student-optimal algorithm and future research should examine the results of the 2 algorithms.^12^ The existing program-centric algorithm has been used for more than 30 years with little examination or comparison to alternative algorithms.

New findings have emerged, and the recruiting environment has changed substantially. The NRMP’s program’s rank list has been found of little value in predicting which residents would do best in residency or would take on academic or leadership roles once graduated.^13^ The general increase in the number of applications per student over time has not been accompanied by increases in match rates^2^ and applicant success, while making the application process less personal.^14^ The residency application process and traditional screening methods have been disrupted by the recent curtailing of educational opportunities and in-person interviews.^15^ The Step 1 exam, which has been used as screening criterion, is becoming pass/fail. The application process needs to improve for applicants and programs by identifying and using predictive factors of resident success in screening, finding better ways to match applicants with programs, and increasing match rates among female applicants and applicants from underrepresented groups.^16^ There is a need for outcomes data for medical educators to reevaluate the process and explore changes.^17^ Incorporating student preferences may be a way of making the recruiting process more personal while also increasing sensitivity to and beginning to meet the expectations of applicants from minority groups. In ranking programs, applicants from underrepresented groups placed a higher weight on patient population, culture, inclusion, and diversity than other students.^18^ Minoritized applicants seem to restrict their search to programs diverse enough for them to feel comfortable.^19^ There is a high prevalence of burnout among trainees, which undermines professional development, places patients at risk, and diminishes well-being.^20^ Permitting a larger role for student preferences in the matching process may improve residents’ fit with program, wellness, trainee experience, and patient care. Incorporating more student preferences may reduce some of the inefficiency in the residency selection process, given that programs and applicants cannot currently discern the true level of interest they have in each other.^15^

This study illustrates the steps in the current program-centric algorithm relative to an alternative student-centric algorithm. It then compares the matching outcomes of the 2 algorithms using simulations of randomized computer-generated students, programs, and positions that reflect NRMP data.

## Methods

Per the Common Rule, this cross-sectional study was exempt from institutional review board or ethics committee review as well as the requirement for informed consent because it did not use actual, private patient, student, or program data.

### Algorithms

The Figure shows the flowcharts of the 2 algorithms, specifying and comparing their steps. The program-centric algorithm, which is based on the study by Gale and Shapley,^21^ is documented and explained in the NRMP’s published video and data.^22,23^

**Figure.**
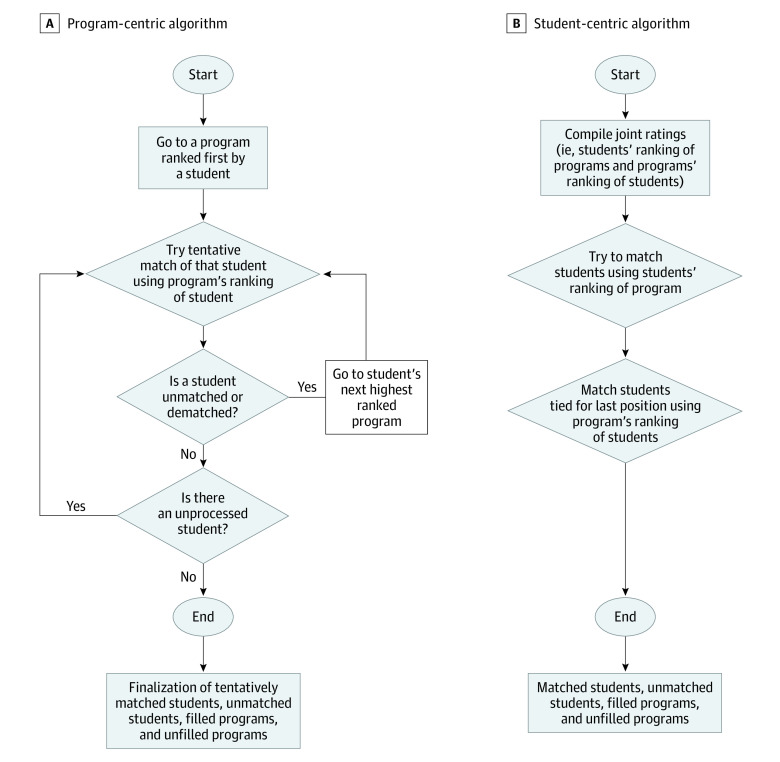
Algorithm Flowcharts

The program-centric algorithm begins by considering an individual student’s proposed, prioritized program. The algorithm then matches using the criterion of the program’s ranking of students. Matches are tentative, in that a student may be dematched in a subsequent iteration after considering other students who are ranked higher by the program. The tentative matches are finalized after the iterative steps consider all students and programs.

The student-centric algorithm starts by compiling joint ratings, ie, student’s rankings of programs and program’s rankings of the student. The matching criteria are applied at the program level. The algorithm considers a student’s ranking of a program first to possibly match to a program’s positions. If students are tied for the program’s last position(s), the program’s ranking of the students is used to break the tie. The algorithm has fewer steps, and its matches are not tentative.

Table 1 illustrates the similarities and differences of the 2 algorithms with an example. Overall, 7 of 8 students (A-H) were selected by 2 programs (designated Alpha and Beta) for interviews. Table 1 lists the rankings students and programs submitted for each other. The program-centric algorithm matched, in order, students D and C to Alpha and students A, F, and E to Beta. The student-centric algorithm matched, in order, students C and B to Alpha and students F, E, and G to Beta. The ties using the initial student’s ranking of a program (ie, students A, B, and C for the 2 positions in Alpha) were broken (in favor of C and B) using the program’s ranking of these tied students.

**Table 1.** Illustration of the 2 Algorithms’ Matches

Student	Student ranking/program ranking	
	Program Alpha, with 2 positions	Program Beta, with 3 positions
A	1/5	2/1^a^
B	1/4^b^	2/7
C	1/2^a^^,^^b^	2/3
D	2/1^a^	1/6
E	2/6	1/4^a^^,^^b^
F	Rank not submitted/3	1/2^a^^,^^b^
G	Not interviewed	1/5^b^
H	1/7	Not interviewed

^a^ Matched using program-centric algorithm.

^b^ Matched using student-centric algorithm.

Some observations of the example are as follows. First, the 2 algorithms matched the same number of students (ie, 5). Next, both algorithms filled all the positions of the 2 programs. Third, the 2 algorithms overlapped for most matches (3 of 5; student C to Alpha; students F and E to Beta). Thus, the 2 algorithms had a common center. Fourth, 2 students (D and A) matched only with the program-centric algorithm, while 2 other students (B and G) matched only with the student-centric algorithm. Fifth, more students matched to their preferred, higher-ranked programs with the student-centric algorithm (all students matched with their first choice) than with the program-centric algorithm (3 students matched with their first choice; 2 students with their second choice). Finally, the last student to match with the program had a better rank in the program-centric algorithm (ranks 2 and 4) than with the student-centric algorithm (ranks 4 and 5).

In the current application system, the student is the least powerful actor.^19^ In the student-centric algorithm, student preferences are given a larger role, but programs do not abdicate all their power. The program would still retain substantial influence through its selection of students for interviews at the beginning of the process, which are needed to submit ranking inputs into the algorithm. At the end of the process, the program would also break any ties using the program’s ranking of students. As more students rank a program highly, the number of ties increases, and the program plays a larger role in breaking ties to determine which of the students match.

### Statistical Analysis

The 2 algorithms were evaluated using the same input data and comparing their matching results. The first step was to generate random data sets based on published NRMP data for 2018, 2019, and 2020. The number of programs and number of students used in each simulation are the same as those published by the NRMP each year and include more than 50 000 students applying to more than 30 000 positions in more than 4000 programs (eTable in the Supplement). For each student, the computer generated a random rank-order list of programs of lengths between 0 and 20. Students were assumed to apply to a single specialty. For each program, the computer generated a randomized rank-order list of students of lengths between 60 and 90. The ratio of the number of students per position was calculated using published NRMP data, and each program was randomly assigned a number of positions around this ratio. This procedure compiled a data set of positions, students, programs, and rank-order lists.

The second step used this data set to generate match results using the program-centric algorithm. The 23 specialties were simulated separately and for each of the years 2018, 2019, and 2020. We used the existing Wilde matching software library^24^ because it is efficiently coded in Python to simulate the results of the match using a program-centric algorithm like the NRMP. The input to the library software was the data set containing the positions, students, and programs and their rank-order lists. The library software ran the program-centric algorithm on the given data and produced the match results.

The third step was to run the student-centric algorithm on the same data set. This simulation was performed using a Python version 3.6 program written by the researchers (K.S.P., B.M., and M.N.K.) that follows the steps shown in the Figure. This type of simulation was not available in Wilde’s software library.

The fourth step was to record the match results from the simulations of the 2 algorithms on the same data set. These 4 steps constituted 1 simulation.

The matching results were averaged across the simulations for each algorithm. We ran a minimum of 1000 simulations each year to attain a margin of error in the results of less than 3%. The following metrics were measured and compared between the 2 algorithms: the proportions of all students who matched, the proportions of students who matched to their first-ranked program overall and in each of 23 specialties, the proportion of students who matched to their top-5–ranked programs, and the rank of the last student matched to a program per position.

It has been posited that a program-optimal and student-optimal algorithm would exhibit little difference.^12^ Accordingly, the null hypotheses were that the 2 algorithms would not be different on these matching outcomes, while the alternative hypotheses were that the 2 algorithms would exhibit differences in these matching outcomes. Analysis was conducted in Excel version 16.49 (Microsoft). Two-tailed tests were performed on these matching outcomes since the student-centric algorithm could possibly have a positive or negative effect. Statistical significance was set at *P* < .05.

## Results

### Overall Match

The overall match percentages for the 2 algorithms for each of the years 2018, 2019, and 2020 are shown in Table 2. No significant differences were observed in overall match rates between the 2 algorithms (eg, for 2020, 59% [95% CI, 57%-61%] vs 58% [95% CI, 56%-60%]; *P* = .73).

**Table 2.** Match Results of 2 Algorithms, by Year, With 2300 Simulations Each Year

Year	Applicants matched				Difference in algorithms	
	Program-centric algorithm		Student-centric algorithm			
	% (95% CI)	No.	% (95% CI)	No.	Difference (95% CI)	*P* value
Overall match						
2020	58.72 (56.71 to 60.73)	35 731	57.84 (55.83 to 59.86)	35 197	−0.88 (−1.97 to 3.73)	.73
2019	59.94 (57.94 to 61.95)	34 903	58.45 (56.44 to 60.47)	34 036	−1.49 (−1.35 to 4.33)	.85
2018	58.97 (56.96 to 60.98)	33 077	57.46 (55.44 to 59.48)	32 230	−1.51 (−1.34 to 4.36)	.85
Student’s first rank						
2020	13.94 (12.53 to 15.36)	8484	50.38 (48.34 to 52.43)	30 656	36.44 (33.95 to 38.92)	<.001
2019	15.69 (14.26 to 17.24)	9134	49.64 (47.59 to 51.68)	28 870	33.89 (31.36 to 36.42)	<.001
2018	15.20 (13.73 to 16.67)	8524	47.66 (45.62 to 49.7)	28 047	32.46 (29.95 to 34.98)	<.001
Student’s top-5 ranks						
2020	46.39 (44.35 to 48.43)	28 227	59.58 (57.57 to 61.59)	36 254	13.19 (10.33 to 16.05)	<.001
2019	50.52 (48.48 to 52.57)	29 347	60.28 (58.32 to 62.32)	35 100	9.80 (6.94 to 12.66)	<.001
2018	50.00 (47.96 to 52.04)	26 735	59.48 (57.47 to 61.49)	33 566	9.48 (6.62 to 12.34)	<.001
Last rank–to-match ratio						
2020	1.91 (1.47 to 2.35)	NA	7.97 (5.86 to 10.08)	NA	−6.06 (−8.21 to −3.9)	<.001
2019	2.25 (1.62 to 2.88)	NA	7.76 (5.75 to 9.78)	NA	−5.51 (−7.63 to −3.4)	<.001
2018	2.27 (1.62 to 2.93)	NA	7.88 (5.67 to 10.09)	NA	−5.6 (−7.91 to −3.3)	<.001

Abbreviation: NA, not applicable.

### Match to Students’ First-Ranked and Top-5–Ranked Programs

The match percentages of the program-centric and student-centric algorithms to students first-ranked and top-5–ranked programs are also shown in Table 2. The student-centric algorithm matched significantly higher percentages of students to both their first-ranked and top-5–ranked programs than the program-centric algorithm (eg, for 2020, first-ranked: 50% [95% CI, 48%-52%] vs 14% [95% CI, 13%-15%]; *P* < .001; top-5–ranked: 60% [95% CI, 58%-62%] vs 46% [95% CI, 44%-48%]; *P* < .001). This finding held in each of the 3 years examined.

### Match to First-Ranked Program in Each of 23 Specialties

Table 3 shows the percentage of students matched to their first-ranked program in each of 23 specialties by the 2 algorithms. The student-centric algorithm matched a significantly higher percentage of students in every specialty. This finding held in each of the 3 years examined.

**Table 3.** Match to Top-Ranked Program in Each of 23 Specialties With 1000 Simulations, 2020

Specialty	Applicants, No.	Applicants matched				Difference in algorithms	
		Program-centric algorithm		Student-centric algorithm			
		% (95% CI)	No.	% (95% CI)	No.	% (95% CI)	*P* value
Other	150	3.13 (2.05 to 4.21)	5	17.39 (15.04 to 19.74)	26	14.26 (11.67 to 16.85)	<.001
Dermatology	264	4.42 (3.15 to 5.69)	12	20.68 (18.17 to 23.19)	55	16.26 (13.44 to 19.08)	<.001
Thoracic surgery	120	3.94 (2.73 to 5.15)	5	29.48 (26.65 to 32.31)	35	25.54 (22.47 to 28.61)	<.001
Physical medicine and rehabilitation	550	6.24 (4.74 to 7.74)	34	28.14 (25.35 to 30.93)	155	21.9 (18.74 to 25.06)	<.001
vascular surgery	153	9.63 (7.8 to 11.46)	15	41.11 (38.06 to 44.16)	63	31.48 (27.92 to 35.04)	<.001
Radiology	1224	4.38 (3.11 to 5.65)	54	15.53 (13.29 to 17.77)	190	11.15 (8.57 to 13.73)	<.001
Child neurology	213	28.41 (25.61 to 31.21)	61	53.92 (50.83 to 57.01)	115	25.51 (21.34 to 29.68)	<.001
Plastic surgery	291	13.69 (11.56 to 15.82)	40	56.05 (52.97 to 59.13)	163	42.36 (38.62 to 46.1)	<.001
Primary medicine	2547	9.07 (7.29 to 10.85)	231	22.49 (19.9 to 25.08)	573	13.42 (10.28 to 16.56)	<.001
Neurological surgery	397	13.53 (11.41 to 15.65)	54	58.59 (55.54 to 61.64)	233	45.06 (41.34 to 48.78)	<.001
Neurology	1226	12.98 (10.9 to 15.06)	159	58.76 (55.71 to 61.81)	720	45.78 (42.09 to 49.47)	<.001
Otolaryngology	505	10.57 (8.66 to 12.48)	53	57.76 (54.7 to 60.82)	292	47.19 (43.58 to 50.8)	<.001
Anesthesiology	2437	18.63 (16.22 to 21.04)	454	57.69 (54.63 to 60.75)	1406	39.06 (35.16 to 42.96)	<.001
Pathology	917	10.34 (8.45 to 12.23)	95	61.48 (58.46 to 64.5)	564	51.14 (47.58 to 54.7)	<.001
Transitional	3801	16.02 (13.75 to 18.29)	609	44.06 (40.98 to 47.14)	1675	28.04 (24.21 to 31.87)	<.001
Orthopedic surgery	1192	17.05 (14.72 to 19.38)	203	71.05 (68.24 to 73.86)	847	54 (50.35 to 57.65)	<.001
Emergency medicine	3473	28.6 (25.8 to 31.4)	993	74.54 (71.84 to 77.24)	2589	45.94 (42.05 to 49.83)	<.001
OB/GYN	2225	17.06 (14.73 to 19.39)	380	70.2 (67.37 to 73.03)	1562	53.14 (49.47 to 56.81)	<.001
Psychiatry	2964	14.16 (12 to 16.32)	420	63.33 (60.34 to 66.32)	1877	49.17 (45.48 to 52.86)	<.001
Pediatrics	5517	24.35 (21.69 to 27.01)	1343	68.12 (65.23 to 71.01)	3758	43.77 (39.84 to 47.7)	<.001
Surgery	4675	11.15 (9.2 to 13.1)	521	59.22 (56.17 to 62.27)	2769	48.07 (44.45 to 51.69)	<.001
Family medicine	7198	17.12 (14.79 to 19.45)	1232	65.98 (63.04 to 68.92)	4749	48.86 (45.11 to 52.61)	<.001
Internal medicine	18 810	26.24 (23.51 to 28.97)	4936	63.21 (60.22 to 66.2)	11 890	36.97 (32.92 to 41.02)	<.001

Abbreviation: OB/GYN, obstetrics/gynecology.

### Rank of Last Student Matched per Position

The programs’ mean of the ratio of the rank of the last student matched in program to its number of positions appear in Table 2. The results show that the program-centric algorithm resulted in last positions going to students with significantly higher program rankings than in the student-centric algorithm (eg, for 2020, 2 [95% CI, 1-2] vs 8 [95% I, 6-10]; *P* < .001). This finding held for each of the 3 years examined.

## Discussion

The current program-centric algorithm has been used by the NRMP to match students to residency positions for a generation. Program directors have been confused about how the match actually works and feel it is too expensive and time-consuming for both applicants and programs.^4^ Students felt it favors programs over applicants and seek an algorithm more favorable to students.^5,7^ The program-centric algorithm matches primarily through the use of the program’s ranking of students, which has recently been found not to be associated with student performance in a residency program, pursuit of academic positions, or leadership achievements.^13^ These factors prompted a comparison of the program-centric algorithm and a student-centric algorithm, in which student ranking of programs is primary and program ranking of students secondary.

A program-optimal algorithm has been posited to exhibit little difference from an applicant-optimal algorithm.^12^ This comparison study found that the 2 algorithms were not different in overall match rates; however, the student-centric algorithm matched more students to their first-ranked and top-5 programs, but it also decreased the program’s ranking of the last matched student compared with the program-centric algorithm.

There is a need to examine how the program’s ranking and student’s ranking criteria relate to postmatch resident and program performance. Recent research has found that the program’s ranking of students is not associated with resident performance.^11^ If a program’s ranking or student’s ranking improves resident and/or program performance, it provides support for its use in screening applications. Research is also needed to analyze if a combination of these 2 rankings improves performance. Another consideration is how the screening criteria should be adapted to a changing environment. As the average number of applications has increased over time, the process has become impersonal.^14^ The process may become more personal by incorporating more students’ preferences in the matching algorithm, including those of minoritized students who are attuned to diversity, inclusion, desired patient population, and atmosphere,^18^ and including preferences of students seeking a personal fit with the program, which may improve resident well-being and patient care.

### Limitations

This study has limitations. It used randomized computer-generated data on positions, programs, students, and rank-ordered lists based on NRMP data. Future research should include real data on these variables and associate them with matching outcomes as well as with longer-term, diverse indicators of resident and program performance. The matching algorithms relied on a single summary ranking by students and programs. Future research should explore inclusion of multiple rankings and variables, such as geographical affinity, to develop richer matching models. This study showed that the 2 algorithms had relative strengths and weaknesses. Future research should explore how algorithms may be combined to build on their strengths and cover their weaknesses. An algorithm change may have varied intended and unintended consequences that should be researched to guide implementation. Further research is also needed on the relative impacts of the 2 algorithms on the number of applications, interviews, and the cost and time demands on students and programs, which have been increasing over time.

## Conclusions

In this study, the current program-centric algorithm, which has been used for 30 years without a comparative evaluation, and a student-centric algorithm were similar in overall match rates, but the student-centric algorithm matched more students to their first-ranked and top-5 programs, while the program-centric algorithm increased a program’s rank of the last matched student. The application environment is changing and disrupting traditional application metrics and methods. Research should examine prospects for increasing inputs in algorithms, the effects of these algorithms on resident and program performance after the match, how to adapt them to a changing environment, and whether biases exist in algorithms that differentially affect participants.

## Notes

Supplement. **eTable. **Number of Programs, Applicants, and Positions in 23 Specialties
