# Supplementary material for: Comparison of Program-centric vs Student-centric National Resident Matching Algorithms
Source: JAMA Netw Open. 2021 Jun 16;4(6):e2113769. doi: 10.1001/jamanetworkopen.2021.13769 (PMC8209592; doi:10.1001/jamanetworkopen.2021.13769)
Supplement: Supplement. — eTable. Number of Programs, Applicants, and Positions in 23 Specialties [file jamanetwopen-e2113769-s001.pdf]

Supplemental Online Content

Mascarenhas B, Puranam KS, Katehakis MN. Comparison of program-centric vs student-centric national resident matching algorithms. *JAMA Netw Open.* 2021;4(6):e2113769. doi:10.1001/jamanetworkopen.2021.13769

eTable. Number of Programs, Applicants, and Positions in 23 Specialties

This supplemental material has been provided by the authors to give readers additional information about their work.

© 2021 Mascarenhas B et al. *JAMA Network Open.*

eTable. Number of Programs, Applicants, and Positions in 23 Specialties

Condensed from: National Resident Matching Program, Results and Data: 2020 Main Residency Match. National Resident Matching Program, Washington, DC. 2020.

	Number of programs	Number of Applicants	Number of Positions
Other	13	150	22
Dermatology	19	264	37
Thoracic Surgery	29	120	38
Physical Medicine & Rehab	39	550	151
Vascular Surgery	59	153	75
Radiology	61	1224	175
Child Neurology	73	213	159
Plastic Surgery	84	291	180
Primary Medicine	85	2547	430
Neurological Surgery	112	397	232
Neurology	126	1226	682
Otolaryngology	129	505	350
Anesthesiology	155	2437	1373
Pathology	164	917	603
Transitional	176	3801	1436
Orthopedic Surgery	203	1192	849
Emergency Medicine	272	3473	2701
OB/GYN	290	2225	1461
Psychiatry	331	2964	1895
Pediatrics	380	5517	3432
Surgery	586	4675	2710
Family Medicine	710	7198	4667
Internal Medicine	952	18810	10608
**Total**	**5048**	**60849**	**34266**

© 2021 Mascarenhas B et al. *JAMA Network Open.*
